# Influence of Genetic Polymorphisms and Biochemical Biomarkers on Response to Nutritional Iron Supplementation and Performance in a Professional Football Team: A Pilot Longitudinal Study

**DOI:** 10.3390/nu17081379

**Published:** 2025-04-19

**Authors:** David Varillas-Delgado

**Affiliations:** 1 Exercise and Sport Science, Faculty of Health Sciences, Universidad Francisco de Vitoria, 28223 Pozuelo, Spain; david.varillas@ufv.es or d.varillas@sportnomics.es; 2 SPORTNOMICS S.L., 28922 Madrid, Spain

**Keywords:** iron supplementation, genetic profile, performance, personalized nutrition, football

## Abstract

Background: Iron deficiency is a prevalent issue among elite athletes, particularly in endurance-based sports like football, where optimal iron status is crucial for aerobic capacity and performance. Despite the well-documented role of iron in oxygen transport and energy metabolism, the interplay between genetic polymorphisms, biochemical markers, and iron supplementation remains poorly understood. This study aimed to investigate the relationship between genetic polymorphisms and iron status in professional football players, assess the impact of iron supplementation on athletic performance, and develop a predictive model for iron supplementation based on genetic and biochemical profiles. Methods: A longitudinal study was conducted over three seasons (2021–2024) with 48 male professional football players. Participants underwent genotyping for polymorphisms in *ACE* (rs4646994), ACTN3 (rs1815739), *AMPD1* (rs17602729), *CKM* (rs8111989), *HFE* (rs1799945), and *MLCK* (rs2700352, rs28497577). Biochemical markers (ferritin, haemoglobin, haematocrit, serum iron) and performance metrics (GPS-derived data) were monitored. Iron supplementation (105 mg/day ferrous sulphate) was administered to players with ferritin <30 ng/mL. A Total Genotype Score (TGS) was calculated to evaluate genetic predisposition. Results: Players with “optimal” genotypes (*ACE* DD, *ACTN3* CC, *AMPD1* CC, *HFE* GC) required less iron supplementation (TGS = 51.25 vs. 41.32 a.u.; *p* = 0.013) and exhibited better performance metrics. Iron supplementation significantly improved haemoglobin and haematocrit in deficient players (*p* < 0.05). The TGS predicted supplementation need (AUC = 0.711; *p* = 0.023), with a threshold of 46.42 a.u. (OR = 5.23, 95% CI: 1.336–14.362; *p* = 0.017 for non-supplemented players). Furthermore, performance data revealed that iron-supplemented players had significantly lower competition time (1128.40 vs. 1972.84 min; *p* = 0.003), total distance covered (128,129.42 vs. 218,556.64 m; *p* = 0.005), and high-speed running in the 18–21 km/h (7.58 vs. 10.36 m/min; *p* = 0.007) and 21–24 km/h (4.43 vs. 6.13 m/min; *p* = 0.010) speed zones. They also started fewer matches (11.50 vs. 21.59; *p* < 0.001). Conclusions: Genetic profile combined with biochemical monitoring effectively predicts iron supplementation needs in athletes. Personalized nutrition strategies, guided by TGS, can optimize iron status and enhance performance in elite football players. This approach bridges a critical gap in sports science, offering a framework for precision nutrition in athletics.

## 1. Introduction

Sports performance results from a complex interplay of genetic, biochemical, and environmental factors. In disciplines such as football and cycling, which demand both aerobic and anaerobic capacities, proper nutrition plays a key role in optimizing athletic performance [1,2].

Iron is an essential mineral for athletic performance, particularly in high-performance athletes such as football players. It plays a crucial role in energy metabolism, oxygen transport, and acid–base balance [3,4]. Many metabolic enzymes depend on iron, including those involved in the citric acid cycle and the electron transport chain, both of which are fundamental for energy production during exercise [5,6,7]. Athletes often require higher iron levels due to the physical demands of training and competition. For instance, it has been suggested that ferritin levels in athletes, particularly those training at altitude, should be higher than standard reference values to optimize performance [3,8]. Iron deficiency, even in the absence of anaemia, can compromise athletic performance. Studies have shown that athletes with low ferritin levels experience reductions in aerobic capacity and increased muscular fatigue [9,10,11]. Moreover, intense exercise can elevate hepcidin levels, a hormone that regulates iron absorption, potentially limiting iron availability and negatively affecting performance [12,13]. Therefore, it is essential for athletes to regularly monitor their iron and ferritin levels and consider strategies such as supplementation or dietary adjustments to maintain optimal levels that support athletic performance [10].

Low serum ferritin levels in athletes (<30 ng/mL) have been consistently identified as a critical factor impairing athletic performance, particularly among high-performance and professional athletes [14]. Scientific literature underscores that ferritin, as a key marker of iron stores, plays a pivotal role in oxygen transport, energy metabolism, and overall endurance capacity [15]. Several studies have demonstrated that athletes with ferritin levels below 30 ng/mL exhibit reduced aerobic capacity, increased fatigue, and suboptimal recovery, even in the absence of overt anaemia [11,13,16,17]. This condition, often referred to as “non-anaemic iron deficiency”, is particularly prevalent in endurance athletes due to factors such as haemolysis, gastrointestinal iron loss, and inadequate dietary intake [18,19,20]. Consequently, supplementation strategies, including oral iron or intravenous administration, have been shown to restore ferritin levels and improve performance metrics [21,22,23]. These findings emphasize the necessity of regular monitoring and targeted intervention to maintain optimal iron status in elite athletes.

Emerging evidence suggests that genetic polymorphisms in genes such as the human homeostatic iron regulator (*HFE*) gene play a critical role in iron homeostasis and may influence individual responses to iron supplementation [24]. Polymorphisms in the *HFE*, particularly c.187C>G; rs1799945 and c.845G>A; rs1800562, are known to significantly influence iron metabolism and absorption, which can have critical implications for professional athletes who often have heightened iron requirements due to intense physical training and increased erythrocyte turnover [25]. The HFE protein, which regulates hepcidin expression, plays a pivotal role in iron homeostasis by modulating the interaction between transferrin and transferrin receptor 1 (*TfR1*) [26]. The c.845G>A; rs1800562 mutation, a common cause of hereditary hemochromatosis, disrupts this regulation, leading to excessive intestinal iron absorption and subsequent iron overload [27], while the c.187C>G; rs1799945 variant, though less severe, can also alter iron metabolism, potentially resulting in suboptimal iron levels or mild iron accumulation [28]. In athletes, these polymorphisms may exacerbate the risk of iron deficiency or toxicity, impacting performance and recovery. For instance, iron deficiency can impair oxygen transport and aerobic capacity, whereas iron overload may increase oxidative stress and inflammation [29]. Previous research has highlighted the clinical relevance of these polymorphisms in iron-related disorders, underscoring the need for further research on their impact in athletic populations [30,31].

The integration of a genetic profile encompassing polymorphisms such as angiotensin converting-enzyme 1 (*ACE* I/D; rs4646994), alpha-actinin 3 (*ACTN3* c.1729C>T; rs1815739), adenosine monophosphate deaminase 1 (*AMPD1* c.34C>T; rs17602729), muscle-specific creatine kinase (*CKM* c.*800A>G; rs8111989), and myosin-like chain kinase (*MLCK* c.49T>C; rs2700352 and c.37885C>A; rs28497577), previously associated with muscle performance, injuries, and recovery [32,33,34], could provide a predictive framework to optimize iron metabolism and prevent low ferritin levels in athletes. Despite these advances, no standardized framework exists to combine genetic, performance, and biochemical data for tailored iron supplementation and dietary recommendations.

Therefore, the aims of this research were (i) to investigate the relationship between genetic polymorphisms and iron status in professional football players, (ii) to assess the impact of iron supplementation on athletic performance, and (iii) to develop a predictive model for iron supplementation based on genetic and biochemical profiles. The author hypothesizes that genetic profile values (specifically those related to iron absorption and muscle performance) combined with biochemical markers (serum ferritin, serum iron, haemoglobin, and haematocrit) can predict the need for supplementation and guide personalized nutrition strategies to optimize football players’ performance.

## 2. Materials and Methods

### 2.1. Participants

A total of 48 male football players from a professional club were included in this pilot longitudinal descriptive study. All participants were active members of a club competing in the Hypermotion division of the Spanish professional football league. Data collection was conducted across three consecutive seasons: 2021–2022, 2022–2023, and 2023–2024.

The baseline demographic characteristics of all football players during the three seasons are presented in Table 1.

The inclusion criteria for this study were defined as follows: (i) professional football players over 18 years of age; (ii) serum ferritin levels below 35 ng/mL; (iii) participation in almost six months in the first team during the season; and (iv) consistent engagement in regular training over the preceding six months. The exclusion criteria included (i) professional football players who had received iron supplementation within three months prior to the study’s initiation; (ii) goalkeepers; and (iii) individuals with disabling traumatic injuries that impeded football training or matches during the six months preceding the study.

All participants provided written informed consent prior to their involvement. The study was conducted in accordance with ethical standards, with approval from the Research Ethics Committee of Francisco de Vitoria University (IRB UFV 32–2020). Participant confidentiality was safeguarded, adhering to the principles outlined in the Declaration of Helsinki (1964, updated in 2013).

### 2.2. DNA Sample Collection and Genotyping

Biological samples were collected across all seasons, with iron supplementation administered in certain cases. Buccal epithelial cells were obtained using SARSTED swabs and preserved at 4 °C until genetic analysis. DNA was extracted at VIVOLabs (Madrid, Spain) using the automated QIACube system (QIAGEN, Venlo, The Netherlands), achieving DNA concentrations between 25 and 40 ng/mL. The extracted DNA was stored in 100 μL aliquots at −20 °C until genotyping.

The analysed polymorphisms included *ACE* (rs4646994), *ACTN3* (rs1815739), *AMPD1* (rs17602729), *CKM* (rs8111989), *HFE* (rs1799945), and *MLCK* (rs2700352 and rs28497577). Genotyping was conducted using single-nucleotide primer extension (SNPE) with the SNaPshot Multiplex Kit (Thermo Fisher Scientific, Waltham, MA, USA), and results were analyzed via capillary electrophoresis on an ABI3500 instrument (Applied Biosystems, Foster City, CA, USA). The bioinformatic interpretation was performed using GeneMapper 5.0 software (Applied Biosystems, Foster City, CA, USA).

The genomic location of each polymorphism is presented in Table 2.

### 2.3. Biochemical Data Recording

Six complete blood analyses were carried out in July (1st), September (2nd), November (3rd), January (4th), March (5th), and May (6th) during 2021–2022, 2022–2023, and 2023–2024 seasons. Blood samples (10 mL) were drawn from the antecubital vein at 8:00 a.m. after a 10–12 h overnight fast, with participants seated comfortably. Collection was performed using ethylenediaminetetraacetic acid (EDTA) vacutainer tubes, while an additional tube without anticoagulant was used for serum iron and serum ferritin quantification.

Blood samples were analyzed for key iron metabolism markers, including serum ferritin (reflecting iron stores), haemoglobin (Hb, oxygen transport capacity), haematocrit (Hct, red blood cell volume), and serum iron (circulating iron levels). These biomarkers were selected based on their established roles in assessing iron status and aerobic performance in athletes [3,8,15]. Ferritin (<30 ng/mL) served as the primary criterion for iron deficiency, while Hb and Hct evaluated oxygen-carrying capacity, critical for endurance [9,16]. Serum iron provided additional insight into iron availability for metabolic processes [4,10]. All assays were performed using standardized clinical protocols to ensure reliability.

### 2.4. Performance Demands

The match demands were assessed using the WIMU PRO™ GPS device (RealtrackSystems S.L., Almería, Spain). The reliability of the device, both within and between units, was found to be acceptable, with intraclass correlation coefficients of 0.65 for the x-coordinate and 0.85 for the y-coordinate in the analyzed systems [37]. Data were processed and extracted using SPRO™ software (version 958; RealtrackSystems, Almería, Spain). The following metrics were included for statistical analysis: competition time (CT; the sum of minutes covered in each match), season total distance (TD; the sum of the distance covered in each match), mean relative total distance of the season (TD/min; the sum of the distance covered in each match divided by the playing time), season high-speed running (HSR, defined as the distance covered at speeds exceeding 18 km/h, 21 km/h, and >24 km/h), and season relative high-speed running for these speeds (HSR/min). To ensure consistency across measurements, each player used the same GPS unit throughout the analysis period. The device was positioned on the upper back 30 min before each session (training or match) and removed immediately after the session concluded. The monitoring of external load during matches was conducted from the start to the end of the game, excluding the warm-up. The number of matches played as starters and total matches played during the season were also recorded.

### 2.5. Iron Supplementation Protocol

All professional football players who were prescribed oral iron supplements did so on an empty stomach of Ferogradumet (Teofarma Srl, Pavia, Italy); 105 mg/day (325 mg/day ferrous sulphate) in the form of fasting tablets, immediately after the results of the first analysis with ferritin values below 35 ng/mL [15,38,39], and were maintained throughout the season until the end of the season in May–June. In addition, all cyclists received Redoxon (Bayer, Leverkusen, Germany) multivitamin pills, that included folic acid (200 μg/day), vitamin C (1000 mg/day), vitamin B12 (1000 μg/day), zinc (10 mg/day), and vitamin D (10 μg/day).

### 2.6. Polygenic Profile of Selected Genes

The combined effect of the seven polymorphisms was evaluated using a Total Genotype Score (TGS), based on the method described by Williams and Folland [40]. A Genotype Score (GS) of 2 was assigned to the “optimal” genotype, a GS of 1 to the heterozygous genotype, and a GS of 0 to the “least favourable” genotype in professional football players, as demonstrated in previous studies [32,34]. The GS scores for the six polymorphisms in the cohort of professional football players are detailed in Table 3.

The GS of all genotypes was converted to a 0–100 arbitrary unit (a.u.) scale to simplify interpretation. This converted score, known as the TGS, was calculated as follows:

TGS = (GS*_ACE_* + GS*_ACTN3_* + GS*_AMPD1_* + GS*_CKM_* + GS*_HFE_* + GS*_MLCK49_* + GS*_MLCK37885_*) × 100/14

### 2.7. Statistical Analysis

Statistical analyses were performed using the Statistical Package for the Social Sciences (SPSS), version 25.0 for Windows (IBM Corp., 2012. IBM SPSS Statistics for Windows, Version 25.0. Armonk, NY: IBM Corp., USA). SNP imbalances were assessed using Hardy–Weinberg Equilibrium (HWE) and the method proposed by Weir and Cockerham [41]. The data are presented as mean ± standard deviation (SD). Normality for continuous variables was assessed using the Shapiro–Wilk test, which was selected due to its superior sensitivity in detecting departures from normality in small sample sizes (n < 50), as in this pilot study (n = 48). For parametric comparisons, independent samples t-tests were used to evaluate differences in physical performance and Total Genotype Scores (TGS) between players who received iron supplementation and those who did not. The significance threshold was set at α = 0.05. Pearson correlation analyses were conducted to assess associations between TGS and GPS-derived performance data.

To evaluate the discriminatory capacity of TGS in predicting iron supplementation status (yes/no), we employed a Receiver Operating Characteristic (ROC) curve analysis [42]. The area under the ROC curve (AUC) was calculated with 95% confidence intervals to determine the sensitivity and specificity of TGS as a predictive tool. This approach enables quantification of the diagnostic performance of the model and is particularly useful when aiming to distinguish between binary outcomes, such as supplementation status.

To further assess the predictive strength of the TGS and its potential application in precision nutrition, a binary logistic regression model was used. This model examined the relationship between TGS (as a continuous predictor) and the likelihood of not requiring iron supplementation (binary outcome), reporting odds ratios (ORs) with 95% confidence intervals.

A three-way analysis of variance (ANOVA; 2 × 2/3 × 6, corresponding to iron supplementation × genotype × biochemistry) was used to compare haemoglobin, haematocrit, serum ferritin, and serum iron during the seasons in predicted polymorphisms to iron supplementation. When a significant F value was obtained for any main effect or interaction, a least significant difference (LSD) post hoc analysis was performed to determine pairwise differences for the values obtained vs. those without supplementation within each genotype.

In all statistical tests, a level of *p* < 0.050 was set to establish statistically significant differences.

## 3. Results

The analyzed polymorphisms met the HWE (all *p* > 0.05; Table 2).

Fourteen football players (29.2%) received iron supplementation during the seasons. Of these, five had supplementation from the first biochemical test in July, four from the biochemical test in January, and five from the biochemical test in March until the end of the season.

Players supplemented with iron made significant differences in performance variables over the three seasons compared to players not supplemented with iron (Table 4).

### 3.1. TGS and Iron Supplementation

When the TGS for all polymorphisms was calculated, the mean TGS for players supplemented with iron was 41.32 a.u. (±13.48 a.u.), with a statistical kurtosis of 0.42 (±0.48). In contrast, players without iron supplementation had a mean TGS of 51.25 a.u. (±11.85 a.u.), with a statistical kurtosis of 0.34 (±0.52). The TGS values were significantly higher in players not supplemented with iron compared to those supplemented with iron (*p* = 0.013).

**Figure 1:**
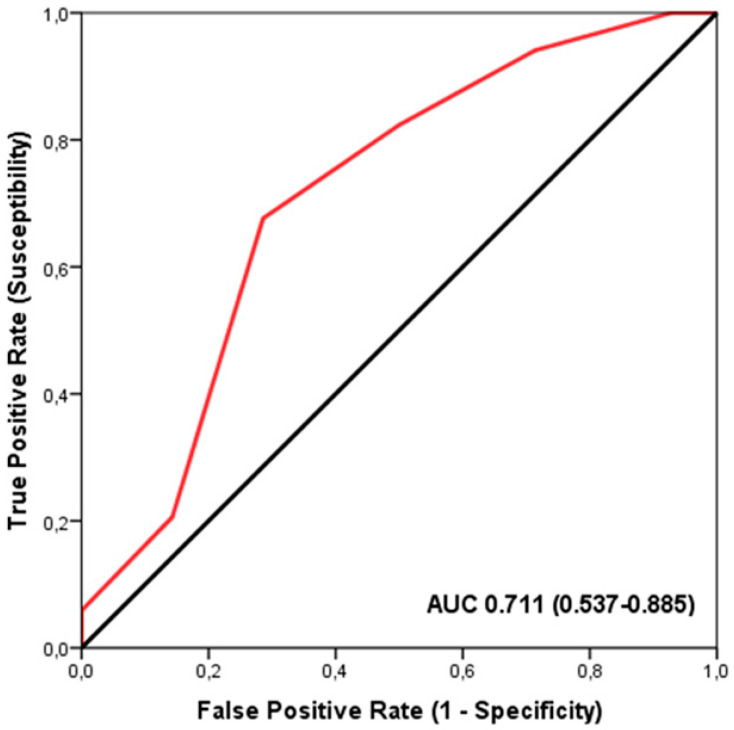
Receiver Operating Characteristic (ROC) curve summarizing the ability of the Total Genotype Score (TGS) to differentiate potential iron supplementation from non-supplemented players in the profile of selected genetic markers. AUC, area under the curve.

The ROC analysis demonstrated a significant discriminatory accuracy of the TGS in identifying players supplemented with iron (AUC = 0.711; 95% CI: 0.537–0.885; *p* = 0.023) (sensitivity = 0.676, specificity = 0.286) (Figure 1). The TGS value corresponding to this threshold was 46.42 a.u. Moreover, binary logistic regression analysis revealed that players with a TGS value above 46.42 a.u. had an odds ratio (OR) of 5.23 (95% CI: 1.336–14.362; *p* = 0.017) for not being supplemented with iron compared to those with a lower TGS value.

The genotypic distribution of polymorphisms in players not supplemented with iron, compared to those supplemented with iron, was statistically significant for the I/D polymorphism of the *ACE* gene (*p* = 0.001). A higher frequency of the “optimal” genotype (DD) was observed in players not supplemented with iron (35.3%) compared to players supplemented with iron (7.1%), whereas players not supplemented with iron exhibited a lower frequency of the “non-optimal” genotype (II) (8.8%) compared to supplemented with iron (57.1%). Similarly, significant differences were noted for the *ACTN3* c.1729C>T polymorphism (*p* = 0.011), where the ‘optimal’ genotype (CC) was more prevalent among players not supplemented with iron (41.2%) versus those supplemented with iron (0.0%). The *AMPD1* c.34C>T polymorphism also showed significant differences (*p* = 0.035), with a higher frequency of the ‘optimal’ genotype (CC) in players not supplemented with iron (85.3%) compared to those supplemented with iron (57.1%). Finally, the *HFE* c.187C>G polymorphism showed significant differences (*p* = 0.011), with a higher frequency in the “non-optimal” genotype (CC) for players supplemented with iron (78.6%) compared to those not supplemented with iron (38.2%). Although the *MLCK* c.49C>T polymorphism did not reach statistical significance, it demonstrated a trend (*p* = 0.080) with a greater frequency of the heterozygous genotype (CT) in players supplemented with iron (57.1%) compared to those not supplemented with iron (23.5%). No differences were observed in other polymorphisms between players supplemented with iron and those not supplemented (Table 5).

Regarding statistical results in genotype distribution among professional football players supplemented with iron and not supplemented, an ANOVA analysis was performed for biochemical markers.

### 3.2. Biochemical Markers and ACE I/D Genotypes

**Figure 2:**
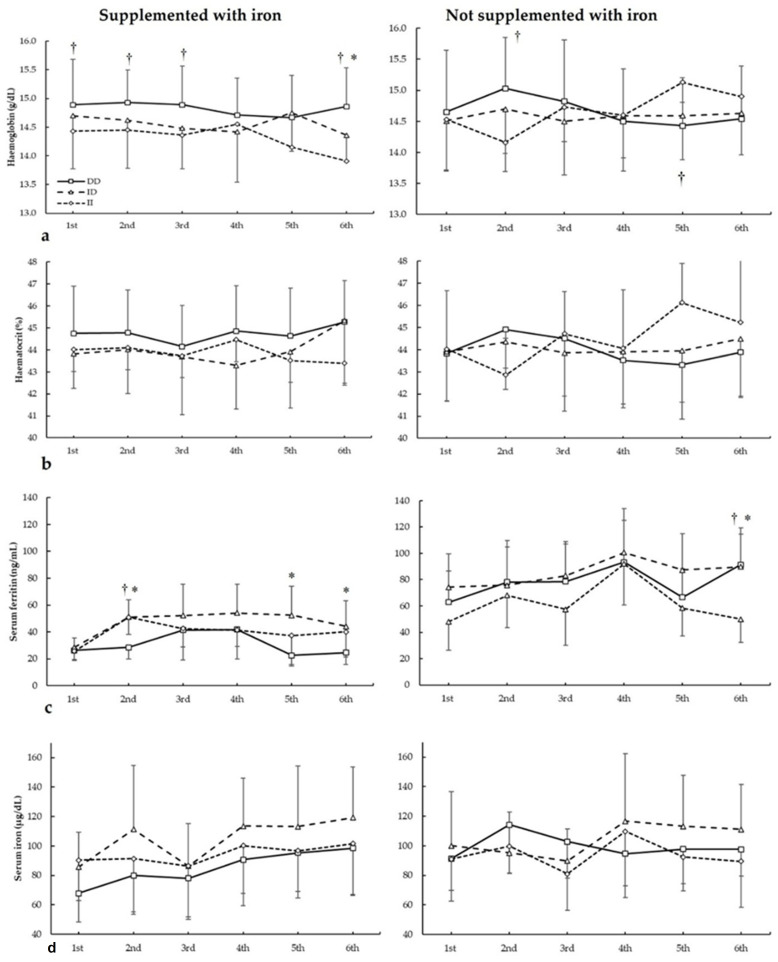
(**a**) Haemoglobin, (**b**) haematocrit, (**c**) serum ferritin, and (**d**) serum iron during whole season bio-chemistries in participants with and without iron supplementation with different ACE I/D (rs4646994) genotypes. * DD genotype is different from ID genotype for the same biochemistry at *p* < 0.050. † DD genotype is different from II genotype for the same biochemistry at *p* < 0.050.

Figure 2 depicts haemoglobin, haematocrit, serum ferritin, and serum iron in biochemistries during the seasons in all three *ACE* I/D genotypes. For haemoglobin (Figure 2a), there were no main effects of the biochemistry (F = 1.844, *p* = 0.169), iron supplementation (F = 0.676, *p* = 0.422), and genotype (F = 0.553, *p* = 0.585). There were statistically significant interactions for these variables on haemoglobin during seasons in biochemistry × genotype (F = 4.085, *p* = 0.34) and supplementation × biochemistry × genotype (F = 7.813, *p* = 0.002). The post hoc analysis revealed that, in comparison to II, DD football players had increased haemoglobin in the first, second, third, and sixth biochemistry in players supplemented with iron (all *p* < 0.050). For players not supplemented with iron, the post hoc analysis revealed that DD football players had higher haemoglobin than II subjects in the second biochemistry (*p* = 0.013).

For haematocrit (Figure 2b), there were no main effects of the biochemistry (F = 1.130, *p* = 0.390), iron supplementation (F = 0.634, *p* = 0.436), and genotype (F = 1.404, *p* = 0.299). There were statistically significant interactions for these variables on haemoglobin during seasons in biochemistry × supplementation (F = 3.135, *p* = 0.042), biochemistry × genotype (F = 5.176, *p* = 0.011), and supplementation × biochemistry × genotype (F = 6.410, *p* = 0.005). The post hoc analysis revealed that, in comparison to II, DD and ID players supplemented with iron had increased haematocrit in the sixth biochemistry (all *p* < 0.050). For players not supplemented with iron, the post hoc analysis revealed that DD players had higher haematocrit than II subjects in the second biochemistry, while in the sixth biochemistry, II football players had higher haematocrit than ID and DD subjects (all *p* < 0.05).

Regarding serum ferritin (Figure 2c), there were main effects of the biochemistry (F = 60.277, *p* < 0.001), iron supplementation (F = 21.159, *p* < 0.001), and genotype (F = 5.679, *p* = 0.013). There were statistically significant interactions for these variables on serum ferritin during seasons in biochemistry × supplementation (F = 5.899, *p* = 0.004) and supplementation × biochemistry × genotype (F = 3.891, *p* = 0.021). The post hoc analysis revealed that, in comparison to DD, ID players supplemented with iron had increased serum ferritin in the second, fifth, and sixth biochemistry, and DD had increased serum ferritin in the second biochemistry than II players (all *p* < 0.050). For players not supplemented with iron, the post hoc analysis revealed that ID players had higher serum ferritin than II players in the third biochemistry and DD players had higher serum ferritin than ID/II players in the sixth biochemistry (all *p* < 0.050).

For serum iron (Figure 2d), there were main effects of the biochemistry (F = 9.713, *p* < 0.001) and genotype (F = 6.985, *p* = 0.006), with no main effects of the iron supplementation (F = 1.007, *p* = 0.393). Additionally, there were statistically significant interactions for these variables on serum iron during seasons in biochemistry × genotype (F = 3.970, *p* = 0.025). The post hoc analysis did not reveal any statistically significant effect of iron supplementation at any biochemistries in all genotypes for haematocrit during all seasons (all *p* > 0.050).

### 3.3. Biochemical Markers and ACTN3 c.1729C>T Genotypes

**Figure 3:**
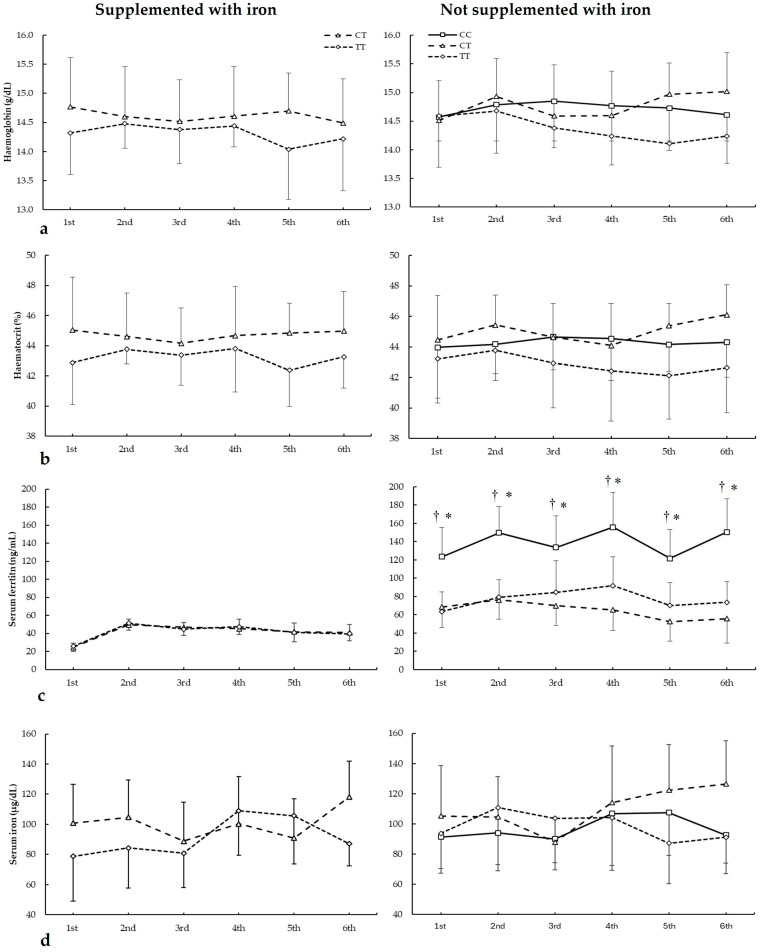
(**a**) Haemoglobin, (**b**) haematocrit, (**c**) serum ferritin, and (**d**) serum iron during whole season biochemistries in participants with and without iron supplementation with different *ACTN3* c.1729C>T (rs1815739) genotypes. * CC genotype is different from CT genotype for the same biochemistry at *p* < 0.050. † CC genotype is different from TT genotype for the same biochemistry at *p* < 0.050.

Figure 3 depicts haemoglobin, haematocrit, serum ferritin, and serum iron in biochemistries during the seasons in all *ACTN3* c.1729C>T genotypes. For haemoglobin (Figure 3a), there were main effects of the genotype (F = 8.340, *p* = 0.018) with no main effects of the biochemistry (F = 3.853, *p* = 0.083) and iron supplementation (F = 0.545, *p* = 0.479). Additionally, there were no statistically significant interactions among these variables on haematocrit during all seasons. The post hoc analysis did not reveal any statistically significant effect of iron supplementation on any biochemistries in all genotypes for haematocrit during all seasons (all *p* > 0.050).

For haematocrit (Figure 3b), there were main effects of the genotype (F = 24.490, *p* < 0.001) with no main effects of the biochemistry (F = 1.300, *p* = 0.390) and iron supplementation (F = 0.111, *p* = 0.747. Additionally, there were no statistically significant interactions among these variables on haematocrit during all seasons. The post hoc analysis did not reveal any statistically significant effect of iron supplementation at any biochemistries in all genotypes for haematocrit during all seasons (all *p* > 0.050).

Regarding serum ferritin (Figure 3c), there were main effects of the biochemistry (F = 14.542, *p* = 0.005), iron supplementation (F = 44.533, *p* < 0.001), and genotype (F = 17.986, *p* < 0.001). There were statistically significant interactions for these variables on serum ferritin during seasons in biochemistry × genotype (F = 5.734, *p* = 0.002), supplementation × genotype (F = 8.678, *p* < 0.001), and supplementation × biochemistry × genotype (F = 11.785, *p* < 0.021). The post hoc analysis revealed that, in comparison to CT and TT, DD players not supplemented with iron had increased serum ferritin in all biochemistries (all *p* < 0.050).

For serum iron (Figure 3d), there were no main effects of the biochemistry (F = 2.436, *p* = 0.175), iron supplementation (F = 2.149, *p* = 0.177), and genotype (F = 3.712, *p* = 0.086). Additionally, there were no statistically significant interactions among these variables on serum iron during all seasons. The post hoc analysis did not reveal any statistically significant effect of iron supplementation at any biochemistries in all genotypes for haematocrit during all seasons (all *p* > 0.050).

### 3.4. Biochemical Markers and AMPD1 c.34C>T Genotypes

**Figure 4:**
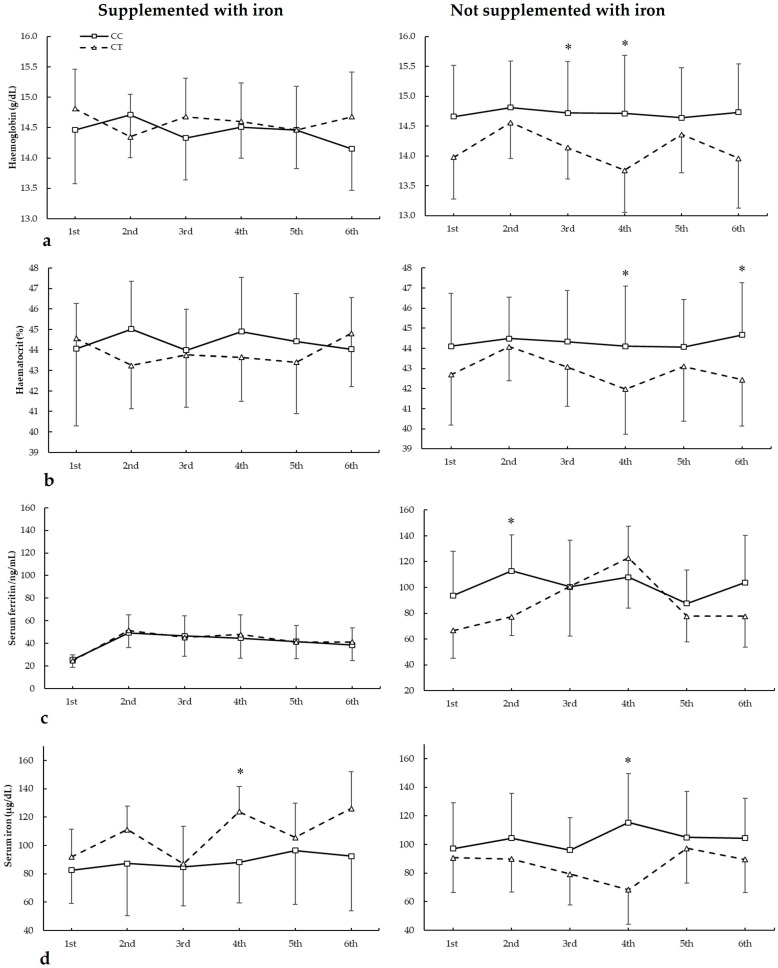
(**a**) Haemoglobin, (**b**) haematocrit, (**c**) serum ferritin, and (**d**) serum iron during whole season biochemistries in participants with and without iron supplementation with different *AMPD1* c.34C>T (rs17602729) genotypes. * CC genotype is different from CT genotype for the same biochemistry at *p* < 0.050.

Figure 4 depicts haemoglobin, haematocrit, serum ferritin, and serum iron in biochemistries during the seasons in all *AMPD1* c.34C>T genotypes. For haemoglobin (Figure 4a), there were main effects of biochemistry (F = 20.205, *p* < 0.001), genotype (F = 50.502, *p* < 0.001), and iron supplementation (F = 80.624, *p* < 0.001). There were statistically significant interactions for these variables on haemoglobin during seasons in biochemistry × supplementation (F = 11.508, *p* < 0.001), biochemistry × genotype (F = 25.073, *p* < 0.001), supplementation × genotype (F = 39.310, *p* < 0.001), and supplementation × biochemistry × genotype (F = 23.532, *p* < 0.001). The post hoc analysis revealed that, in comparison to CT, CC players not supplemented with iron had increased haemoglobin in the third and fourth biochemistries (all *p* < 0.050).

For haematocrit (Figure 4b), there were no main effects of biochemistry (F = 1.994, *p* = 0.116), iron supplementation (F = 3.043, *p* = 0.092), and genotype (F = 3.453, *p* = 0.074). There were statistically significant interactions for these variables on haematocrit during seasons in biochemistry × supplementation (F = 5.507, *p* = 0.002), biochemistry × genotype (F = 3.477, *p* = 0.017), and supplementation × biochemistry × genotype (F = 19.091, *p* < 0.001). The post hoc analysis revealed that, in comparison to CT, CC players not supplemented with iron had increased haematocrit in the fourth and sixth biochemistries (all *p* < 0.050).

Regarding serum ferritin (Figure 4c), there were main effects of the biochemistry (F = 58.514, *p* < 0.001) and iron supplementation (F = 36.030, *p* < 0.001), with no main effects of the genotype (F = 0.367, *p* = 0.551). There were statistically significant interactions for these variables on serum ferritin during seasons in biochemistry × supplementation (F = 5.586, *p* = 0.001), biochemistry × genotype (F = 4.393, *p* = 0.006), and supplementation × biochemistry × genotype (F = 5.903, *p* = 0.001). The post hoc analysis revealed that, in comparison to CT, CC players not supplemented with iron had increased serum ferritin in the second biochemistry (*p* = 0.041).

For serum iron (Figure 3d), there were main effects of the biochemistry (F = 5.791, *p* = 0.001) with no main effects of the iron supplementation (F = 0.548, *p* = 0.465) and genotype (F = 0.018, *p* = 0.896). There were statistically significant interactions for these variables on serum ferritin during seasons in biochemistry × supplementation (F = 3.343, *p* = 0.020), biochemistry × genotype (F = 3.035, *p* = 0.029), supplementation × genotype (F = 26.743, *p* < 0.001), and supplementation × biochemistry × genotype (F = 6.753, *p* < 0.001). The post hoc analysis revealed that, in comparison to CT, CC players not supplemented with iron had increased serum iron in the fourth biochemistry (*p* = 0.018), similar to players supplemented with iron in the fourth biochemistry (*p* = 0.003).

### 3.5. Biochemical Markers and HFE c.187C>G Genotypes

**Figure 5:**
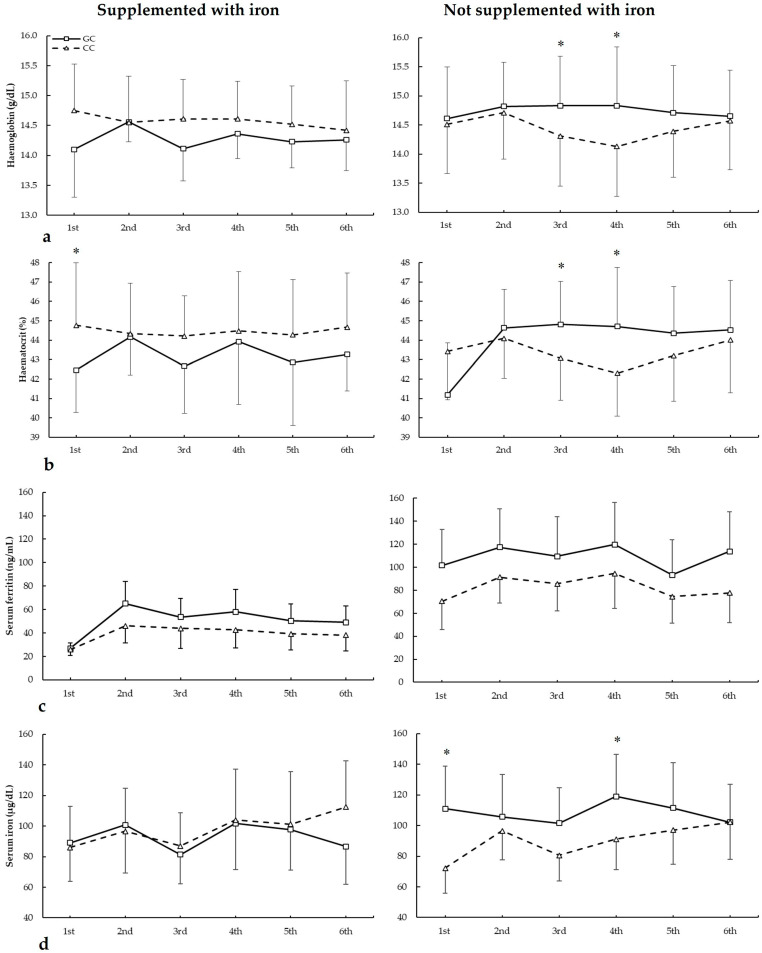
(**a**) Haemoglobin, (**b**) haematocrit, (**c**) serum ferritin, and (**d**) serum iron during whole season biochemistries in participants with and without iron supplementation with different *HFE* c.187C>G (rs1799945) genotypes. * GC genotype is different from CC genotype for the same biochemistry at *p* < 0.050.

Figure 5 depicts haemoglobin, haematocrit, serum ferritin, and serum iron in biochemistries during the seasons in all *HFE* c.187C>G genotypes. For haemoglobin (Figure 5a), there were no main effects of biochemistry (F = 1.404, *p* = 0.275), iron supplementation (F = 2.381, *p* = 0.138), genotype (F = 3.264, *p* = 0.069), and iron supplementation (F = 0.933, *p* = 0.342). There were statistically significant interactions for these variables on haemoglobin during seasons in biochemistry × genotype (F = 2.966, *p* = 0.044), supplementation × genotype (F = 6.505, *p* = 0.019), and supplementation × biochemistry × genotype (F = 2.929, *p* = 0.045). The post hoc analysis revealed that, in comparison to CC, GC players not supplemented with iron had increased haemoglobin in the third and fourth biochemistries (all *p* < 0.050).

For haematocrit (Figure 5b), there were no main effects of biochemistry (F = 2.452, *p* = 0.079), iron supplementation (F = 0.094, *p* = 0.762), and genotype (F = 0.115, *p* = 0.738). However, there were statistically significant interactions for these variables on haematocrit during seasons in biochemistry × supplementation (F = 2.936, *p* = 0.046) and supplementation × genotype (F = 8.875, *p* = 0.007). The post hoc analysis revealed that, in comparison to CC, GC players not supplemented with iron had increased haematocrit in the third, fourth, and sixth biochemistries. Also, CC players supplemented with iron had higher haematocrit than GC in the first biochemistry (all *p* < 0.050).

Regarding serum ferritin (Figure 5c), there were main effects of the biochemistry (F = 22.294, *p* < 0.001) and iron supplementation (F = 17.779, *p* < 0.001), with no main effects of the genotype (F = 3.154, *p* = 0.083). Additionally, there was a statistically significant interaction for these variables on serum ferritin during seasons in biochemistry × supplementation (F = 3.185, *p* = 0.035). The post hoc analysis did not reveal any statistically significant effect of iron supplementation at any biochemistries in all genotypes for serum ferritin during all seasons (all *p* > 0.050).

For serum iron (Figure 5d), there were main effects of the biochemistry (F = 5.319, *p* = 0.005) and supplementation (F = 7.545, *p* = 0.012), with no main effects of genotype (F = 2.586, *p* = 0.123). Additionally, there were statistically significant interactions for these variables on serum ferritin during seasons in biochemistry × genotype (F = 3.104, *p* = 0.042), supplementation × genotype (F = 38.890, *p* < 0.001), and supplementation × biochemistry × genotype (F = 9.023, *p* = 0.007). The post hoc analysis revealed that, in comparison to CC, GC players not supplemented with iron had increased serum iron in the first and fourth biochemistries (all *p* < 0.050).

## 4. Discussion

This is the first study to investigate the relationship between several polymorphisms of muscle performance-related genes and the association between iron supplementation in football players and the likelihood of performance. The present study provides valuable insights into the influence of genetic polymorphisms and biochemical biomarkers on the response to iron supplementation and athletic performance in professional football players. The findings highlight the importance of integrating genetic and biochemical data to optimize nutritional strategies and enhance performance in elite athletes. This research contributes to the growing body of evidence supporting the role of personalized nutrition in sports science, particularly in addressing iron deficiency, a common issue among high-performance athletes.

These findings suggest a significant association between the TGS and the requirement for iron supplementation, indicating that genetic background may influence iron metabolism and individual iron needs in elite athletes. However, the mechanisms linking specific genotypes to iron handling and supplementation efficacy deserve further attention. The *ACE* gene, particularly the I/D (rs4646994) polymorphism, has been linked to variations in cardiovascular efficiency and muscle oxygenation. The D allele is associated with increased ACE activity, which can modulate vasoconstriction and potentially impact iron delivery to muscle tissues during exercise. Higher ACE activity has also been related to enhanced erythropoiesis under hypoxic conditions, potentially influencing iron metabolism and red blood cell production [43,44,45].

Similarly, the c.1729C>T (rs1815739) polymorphism of the *ACTN3* gene plays a role in muscle fibre composition and energy metabolism. The C allele is associated with fast-twitch fibre dominance and higher anaerobic performance, while the T allele is linked to improved endurance capacity and more efficient oxidative metabolism [46,47]. These differences may alter iron demands, as aerobic metabolism and mitochondrial function are closely tied to iron-containing proteins such as cytochromes. Individuals with genotypes favouring endurance (TT genotype) may experience increased iron turnover due to higher mitochondrial density and oxidative stress, increasing susceptibility to iron deficiency under intense training [48].

Moreover, other genes involved in iron metabolism, such as *TMPRSS6*, *HFE*, and *TF*, may influence an athlete’s response to iron supplementation. For instance, variants in the *TMPRSS6* gene can result in elevated hepcidin levels, limiting iron absorption even in the presence of supplementation [49]. Similarly, mutations in *HFE* can affect iron uptake regulation and homeostasis, potentially predisposing athletes to either iron overload or deficiency [50,51]. These genetic differences may explain why some athletes respond poorly to standard oral iron supplementation strategies.

These insights support the potential utility of TGS as a predictive tool in precision sports nutrition, helping identify athletes at greater risk of iron-related imbalances or poor response to supplementation. Future longitudinal studies should investigate how genotype-guided interventions influence long-term athletic performance, recovery, and health outcomes.

### 4.1. Iron Supplementation

Commonly used supplements in football and other sports include caffeine, creatine, iron, protein, carbohydrate and electrolyte drinks, tart cherry juice, beetroot juice (rich in nitrates), sodium bicarbonate with minerals, yohimbine, and proprietary nutraceuticals [52,53,54]. These supplements are widely believed to enhance athletic performance by improving energy metabolism, muscle recovery, endurance, and cognitive function. However, their use may also introduce confounding variables that could affect study outcomes, particularly in research settings [53,55]. The use of ergogenic supplements continues to rise annually across both amateur and professional sports, with a particularly high incidence in football. The effectiveness of these supplements is influenced by a combination of the player’s intrinsic factors and environmental conditions [56]. In addition to these, iron supplementation is frequently utilized, especially among athletes at risk of iron deficiency or iron deficiency nonanemic, such as endurance athletes, female athletes, or those with dietary restrictions [3,10,57]. Supplementation with iron (e.g., ferrous sulphate or ferrous diglycine) has been shown to improve haemoglobin levels, aerobic capacity, and exercise performance in individuals with iron deficiency [58]. However, excessive iron intake can lead to adverse effects, including gastrointestinal distress and oxidative stress [59,60].

Overall, while these supplements, including iron, have the potential to enhance football performance, their efficacy and safety depend on factors such as dosage, timing, and the individual’s physiological needs, highlighting the importance of individualized dosing and monitoring of iron status through biomarkers like serum ferritin and haemoglobin [61]. Further research is needed to optimize their use and minimize potential risks. Consequently, evidence regarding the efficacy of dietary supplements for improving football performance remains mixed, limited, or sometimes lacking altogether [53]. This study highlights that iron supplementation significantly improved performance metrics, particularly in players with low ferritin levels (<30 ng/mL). Supplemented players showed increased haemoglobin and haematocrit levels, which are critical for oxygen transport and aerobic capacity [8]. These findings align with previous research indicating that iron deficiency, even in the absence of anaemia, can impair athletic performance by reducing aerobic capacity and increasing fatigue [9,10]. The results also support the notion that maintaining optimal iron levels is essential for sustaining high-performance levels in athletes, particularly in endurance-based sports like football. The findings of this study are consistent with previous research that has highlighted the importance of iron status in athletic performance. For instance, Nabeyama et al. found that iron-deficient non-anaemic athletes exhibited reduced performance metrics, which improved following iron supplementation [11]. Similarly, Dugan et al. assess the efficacy of both oral and intravenous iron supplementation on physical capacity, quality of life, and fatigue scores in iron-deficient non-anaemic individuals using individual patient data in a systematic review and meta-analysis [62]. The current investigation suggests that genetics may constitute an important contributing factor for a player’s predisposition to iron supplementation. By evaluating a TGS that focuses on just seven polymorphisms linked to muscle performance, it is possible to identify players who may have a greater predisposition to requiring iron supplementation. These athletes can then be integrated into specialized programs designed to address their inherent susceptibility, which could influence performance throughout the football season. The genetic insights provided by this study offer a novel approach to optimizing and personalizing supplementation strategies for professional football players, complementing established nutritional protocols. Beyond its relevance to clinical practice, this finding also paves the way for future research opportunities.

The data presented in Table 4 demonstrate changes in athletic performance indicators following iron supplementation, notably in high-speed running (HSR) metrics and match starting appearances. In football players not supplemented with iron, HSR showed a statistically significant increase in relative HSR 18–21 km/h (*p* = 0.007) and relative HSR 21–24 km/h (*p* = 0.010), indicating an improvement in players’ high-intensity locomotor capacity, which is strongly linked to iron status and oxygen transport efficiency [63,64]. Similarly, the increase in match starting appearances was statistically significant (*p* < 0.001), suggesting that players with improved iron status were more frequently selected to start matches, reflecting better physical readiness and reduced fatigue. These findings support previous literature indicating that correcting iron deficiency can lead to perceptible enhancements in both objective and coach-perceived performance [65]. By confirming statistical significance, these results reinforce the importance of individualized iron monitoring and targeted supplementation in professional football players.

### 4.2. Genetic Profile and Iron Supplementation

Excellent muscle performance in elite athletes is facilitated by an optimal polygenic profile as previously shown [32,33,34,66]. However, the methodological rigor and evidence in genetic association research in football still have room for improvement [67], especially in supplementation [68]. This investigation indicates that the combined influence of these polymorphisms is strong enough to link the response after iron supplementation to muscle performance. Previous research suggests that iron supplementation can enhance strength, power, and work capacity during short, high-intensity efforts, potentially leading to improved performance on the field [69,70]. Additionally, iron absorption may be influenced by genetic markers, which could impact individual responses to supplementation, and it is important to note that the effects of iron supplementation may vary depending on an individual’s genetics [71,72,73]. Different genetic markers can affect the absorption, transport, and regulation of iron in the body, impacting its availability and effectiveness in improving performance. For example, variants in genes such as *HFE*, Transmembrane protease, serine 6 (*TMPRSS6*), or transferrin (*TF*) can alter iron homeostasis [74,75,76], determining an individual’s need for supplementation and its effects on energy metabolism and muscle function.

The identification of genetic markers associated with iron supplementation and the regulation of energy metabolism in skeletal muscles can help sports nutritionists and coaches to develop personalized strategies to adapt the nutritional strategies of supplementation protocols according to the professional football player’s genetic profile, previously presented [34,68]. Therefore, understanding an athlete’s genetic profile can be crucial for optimizing iron supplementation, preventing both deficiencies that could impair performance and excesses that may cause adverse effects.

Moreover, the study’s focus on genetic polymorphisms such as *HFE* c.187C>G and *ACE* I/D aligns with emerging research on the role of genetics in sports performance. Semenova et al. found that the *HFE* c.187C>G polymorphism was associated with endurance athlete status and aerobic capacity, suggesting that genetic variations in iron metabolism may influence athletic performance [29]. Another study by Chicharro et al. indicates a high prevalence of *HFE* gene mutations in endurance athletes compared with sedentary controls, without association in the athletes between *HFE* gene mutations and blood iron markers [77]. However, Varillas-Delgado showed recently that blood biochemical iron metabolism parameters, especially haemoglobin and haematocrit, could be related to performance with the GC genotype of the *HFE* c.186C>G polymorphism [58]. The present study extends this research by demonstrating that these polymorphisms can also predict the need for iron supplementation, offering a potential tool for personalized nutrition in athletes.

### 4.3. Association of TGS with Performance in Professional Football Players

Indeed, the TGS of these polymorphisms related to muscle performance suggests that the likelihood of iron supplementation in professional football players was linked to their performance. Iron supplementation presented a higher risk in the TGS <46.42 a.u. football players than in their >46.42 a.u. (Figure 1). This information from genetic profile studies using TGS should be integrated into the field of genetics in the future, as it provides more comprehensive data for defining phenotypes in the context of nutrition and supplementation optimization [58,68], and even performance [33,78,79], than previous monogenic studies [44,46,77]. The results of this study will allow for specific programs for the individualization of nutrition and iron supplementation strategies, and the use of this genetic profile deserves further investigation.

Football players who required iron supplementation demonstrated significantly lower performance metrics, as assessed by GPS tracking (Table 4), compared to those who did not receive supplementation. These athletes exhibited reduced total distance covered, lower high-speed running capacity, and fewer matches as starters, suggesting an impairment in aerobic capacity and endurance. The findings highlight the crucial role of iron status in sustaining optimal performance levels, and iron status should be carefully monitored during the various training and competitive periods in elite athletes [80]. By integrating genetic profiling into nutritional assessments, it is possible to identify individuals predisposed to iron deficiency due to genetic polymorphisms influencing iron metabolism. The implementation of personalized nutrition strategies based on genetic data could prevent performance disparities by ensuring early intervention and optimized supplementation protocols. This study underscores the necessity of a precision medicine approach in sports science to mitigate the effects of iron deficiency on elite athletes’ performance.

### 4.4. Limitations

Despite its contributions, this study has several limitations: (i) the sample size was relatively small, which may limit the generalizability of the findings. Future research should aim to replicate these findings in larger cohorts, including athletes from different sports and ethnic backgrounds, (ii) the study focused on male football players, and the findings may not be applicable to female athletes or other sports, (iii) although iron supplementation and performance metrics were closely monitored, the study did not comprehensively assess dietary intake, which can significantly influence iron status. Variations in the consumption of iron-rich foods, vitamin C (which enhances absorption), or substances like calcium and polyphenols (which inhibit absorption) were not controlled and could have confounded the results, (iv) the study’s longitudinal design, while a strength, may have been influenced by external factors such as changes in training load, which can impact iron metabolism through increased haemolysis, sweating, and inflammation, was not quantified in detail. This may have introduced interindividual variability in iron demands and supplement responsiveness, and (v) another important limitation of the current study is its exclusive focus on male football players, which restricts the applicability of the findings to athletes of other genders and sporting disciplines. Gender differences play a critical role in iron metabolism, with female athletes often facing higher risks of iron deficiency due to menstrual blood loss, hormonal influences, and generally lower dietary iron intake. Additionally, the type of sports practice can significantly influence iron requirements and metabolism. For instance, endurance athletes typically experience greater iron losses through hemolysis, sweating, and gastrointestinal bleeding compared to strength or power athletes. These physiological and sport-specific differences may affect how genotypes interact with iron status and the need for supplementation. Therefore, the current findings should be interpreted with caution and are not directly generalizable to female athletes or those engaged in sports with different metabolic demands. Future studies should aim to include more diverse cohorts encompassing both male and female athletes from a variety of sports to better understand how genotype, gender, and sport type collectively influence iron metabolism and supplementation strategies. Furthermore, the integration of additional omics data (e.g., transcriptomics and metabolomics) could enhance the predictive power of models like the TGS, offering even more precise nutritional recommendations.

The findings of this study have important practical implications for nutritionists and physical trainers working with elite athletes. Regular monitoring of iron status, combined with genetic testing, can help identify athletes at risk of iron deficiency and allow for early intervention. Additionally, the study highlights the potential benefits of personalized supplementation plans tailored to an athlete’s genetic profile, which can optimize performance and reduce the risk of iron-related deficiencies. This study represents a significant advancement in the field of sports nutrition by integrating genetic and biochemical data to develop a predictive model for iron supplementation. While previous research has primarily focused on isolated biochemical markers, such as serum ferritin and haemoglobin levels, this study incorporates genetic polymorphisms to provide a more holistic approach to athlete management. The use of the TGS to predict iron supplementation status is a novel contribution to the field, offering a potential framework for personalized nutrition strategies in elite athletes, especially professional football players.

## 5. Conclusions

This study is the first to demonstrate a clear association between specific genetic polymorphisms (*ACE* I/D, *ACTN3* c.1729C>T, *AMPD1* c.34C>T, and *HFE* c.187C>G) and the need for iron supplementation. Players with “optimal” genotypes in *ACE* (DD) and *ACTN3* (CC) were less likely to require iron supplementation, suggesting that genetic variations play a significant role in iron metabolism and absorption. This finding underscores the importance of integrating genetic profiling into routine football players’ assessments to identify individuals at risk of iron deficiency and to tailor supplementation strategies accordingly. The results confirmed that iron supplementation significantly improved key performance metrics, such as haemoglobin and haematocrit levels, in players with low ferritin levels (<30 ng/mL). These improvements are critical for oxygen transport and aerobic capacity, which are essential for sustaining high performance in endurance-based sports like football. The study supports the notion that maintaining optimal iron levels through targeted supplementation can enhance athletic performance, particularly in athletes with heightened iron demands due to intense training and competition schedules.

The development of TGS represents a significant advancement in the field of sports nutrition. The TGS, which integrates genetic and biochemical data, demonstrated a strong ability to predict the need for iron supplementation. This tool provides a novel framework for personalized nutrition, enabling nutritionists and physical trainers to identify athletes who may benefit from iron supplementation based on their genetic and biochemical profiles. The TGS also highlights the potential of multi-omics approaches in sports science, paving the way for more precise and individualized nutritional interventions.

## Figures and Tables

**Table 1:** Baseline characteristics of professional football players.

		Professional Football Players, n = 48
	Age, mean (SD)	25.21 (4.32)
	Weight, mean (SD)	70.62 (6.74)
	Height, mean (SD)	178.42 (5.52)
	BMI, mean (SD)	22.21 (1.74)
	Defenders, n (%)	18 (37.5)
Position	Midfields, n (%)	13 (27.1)
	Forwards, n (%)	17 (35.4)

SD, standard deviation.

**Table 2:** Genomic location and minor allele frequency (MAF) of selected genes.

Symbol	Gene	dbSNP	Genomic Location	MAF Football Players	MAF (IBS) *	HWE	FIS
*ACE*	Angiotensin-converting enzyme	rs4646994	17q23.3	47.9% (I)	42.1% (I) **	*p* = 0.252	−0.18
*ACTN3*	Alpha-actinin-3	rs1815739	11q13.2	51.0% (T)	43.9% (T)	*p* = 0.156	−0.31
*AMPD1*	Adenosine monophosphate deaminase 1	rs17602729	1p13.2	7.7% (T)	11.5% (T)	*p* = 0.412	0.15
*CKM*	Muscle-specific creatine kinase	rs8111989	19q13.32	28.1% (G)	26.6% (G)	*p* = 0.893	−0.04
*HFE*	Homeostatic iron regulator	rs1799945	6p21.3	25.0% (G)	25.2% (G)	*p* = 0.963	0.01
*MLCK*	Myosin Light Chain Kinase	rs2700352	3q21.1	27.1% (T)	20.1% (T)	*p* = 0.215	−0.31
	Myosin Light Chain Kinase	rs28497577	3q21.1	18.8% (A)	10.3% (A)	*p* = 0.196	−0.40
*Overall SNPs*						*p* = 0.441	−0.16

IBS, Iberian population in Spain * [35] ** [36]; FIS, inbreeding coefficient; HWE, Hardy–Weinberg equilibrium; MAF, minor allele frequency; SNP, single-nucleotide polymorphism.

**Table 3:** Genotype distribution in professional football players.

Symbol	Gene Name	Polymorphism	dbSNP	Genotype Score	Professional Football Players
*ACE*	Angiotensin-converting enzyme	I/D	rs4646994	2 = DD	13 (27.1%)
				1 = ID	24 (50.0%)
				0 = II	11 (22.9%)
*ACTN3*	Alpha-actinin-3	c.1729C>T	rs1815739	2 = CC	14 (29.1%)
				1 = CT	19 (3.6%)
				0 = TT	15 (31.3%)
*AMPD1*	Adenosine monophosphate deaminase 1	c.34C>T	rs17602729	2 = CC	37 (77.1%)
				1 = CT	11 (22.9%)
				0 = TT	0 (0.0%)
*CKM*	Muscle-specific creatine kinase	c.*800A>G	rs8111989	2 = GG	4 (8.3%)
				1 = GA	19 (39.6%)
				0 = AA	25 (52.1%)
*HFE*	Homeostatic iron regulator	c.187C>G	rs1799945	2 = GG	0 (0.0%)
				1 = GC	24 (50.0%)
				0 = CC	24 (50.0%)
*MLCK*	Myosin Light Chain Kinase	c.49C>T	rs2700352	2 = CC	27 (56.3%)
				1 = CT	16 (33.3%)
				0 = TT	5 (10.4%)
	Myosin Light Chain Kinase	c.37885C>A	rs28497577	2 = AA	1 (2.1%)
				1 = CA	16 (33.3%)
				0 = CC	31 (64.6%)

**Table 4:** Effects of iron supplementation on physical performance for GPS-derived variables in professional football players during the season.

Performance GPS Variables	Supplemented with Iron	Not Supplemented with Iron	*p*-Value
CT (min)	1128.40 (353.78)	1972.84 (346.77)	0.003
TD (m)	128,129.42 (23,677.22)	218,556.64 (74,688.26)	0.005
TD/min (m/min)	112.67 (9.11)	111.81 (8.13)	0.750
HSR (km/h)	32.97 (0.79)	32.83 (1.12)	0.666
Relative HSR 18–21 km/h (m/min)	7.58 (2.31)	10.36 (3.53)	0.007
Relative HSR 21–24 km/h (m/min)	4.43 (1.75)	6.13 (2.53)	0.010
Relative HSR > 24 km/h (m/min)	3.12 (1.37)	3.61 (1.22)	0.256
Matches as starter	11.50 (7.49)	21.59 (6.75)	<0.001
Matches played	21.50 (11.82)	27.59 (11.25)	0.114

CT, competition time; HSR, high-speed running; TD, total distance.

**Table 5:** Genotype distribution among professional football players supplemented with iron and those not supplemented with iron.

Symbol	Gene	Polymorphism	dbSNP	Genotype Score	Supplemented with Iron	Not Supplemented with Iron	*p*-Value
*ACE*	Angiotensin-converting enzyme	I/D	rs4646994	2 = DD	1 (7.1%) ↓	12 (35.3%) ↑	0.001
				1 = ID	5 (35.7%)	19 (55.9%)	
				0 = II	8 (57.1%) ↑	3 (8.8%) ↓	
*ACTN3*	Alpha-actinin-3	c.1729C>T	rs1815739	2 = CC	0 (0.0%) ↓	14 (41.2%) ↑	0.011
				1 = CT	9 (64.3%) ↑	10 (29.4%) ↓	
				0 = TT	5 (35.7%)	10 (29.4%)	
*AMPD1*	Adenosine monophosphate deaminase 1	c.34C>T	rs17602729	2 = CC	8 (57.1%) ↓	29 (85.3%) ↑	0.035
				1 = CT	6 (42.9%) ↑	5 (14.7%) ↓	
*CKM*	Muscle-specific creatine kinase	c.*800A>G	rs8111989	2 = GG	1 (7.1%)	3 (8.8%)	0.950
				1 = GA	6 (42.9%)	13 (38.2%)	
				0 = AA	7 (50.0%)	18 (52.9%)	
*HFE*	Homeostatic iron regulator	c.187C>G	rs1799945	1 = GC	3 (21.4%) ↓	21 (61.8%) ↑	0.011
				0 = CC	11 (78.6%) ↑	13 (38.2%) ↓	
*MLCK*	Myosin Light Chain Kinase	c.49C>T	rs2700352	2 = CC	5 (35.7%)	22 (64.7%)	0.080
				1 = CT	8 (57.1%) ↑	8 (23.5%) ↓	
				0 = TT	1 (7.1%)	4 (11.8%)	
	Myosin Light Chain Kinase	c.37885C>A	rs28497577	2 = AA	0 (0.0%)	1 (2.9%)	0.800
				1 = CA	5 (35.7%)	11 (32.4%)	

↑, statistical higher frequency; ↓, statistical lower frequency.

## Data Availability

The original contributions presented in this study are included in the article. Further inquiries can be directed to the corresponding author.
